# Alternative or cytochrome? Respiratory pathways in traps of aquatic carnivorous bladderwort *Utricularia reflexa*

**DOI:** 10.1080/15592324.2022.2134967

**Published:** 2022-10-20

**Authors:** Andrej Pavlovič, Jana Jakšová, Martin Hrivňacký, Lubomír Adamec

**Affiliations:** a Department of Biophysics, Faculty of Science, Palacký University, Olomouc, Czech Republic; b Department of Experimental and Functional Morphology, Institute of Botany of the Czech Academy of Sciences, Třeboň, Czech Republic

**Keywords:** Alternative oxidase, bladderwort, carnivorous plants, cytochrome *c* oxidase, light harvesting antennae, respiration

## Abstract

Carnivorous plants of the genus *Utricularia* (bladderwort) form modified leaves into suction bladder traps. The bladders are metabolically active plant tissue with high rates of mitochondrial respiration (R_D_). In general, plants possess two mitochondrial electron transport pathways to reduce oxygen to water: cytochrome and an alternative. Due to the high metabolic rate in the bladders, it is tempting to assume that the bladders prefer the cytochrome *c* oxidative pathway. Surprisingly, we revealed that alternative oxidase (AOX), which yields only a little ATP, is much more abundant in the bladders of *Utricularia reflexa* in comparison with the shoots. This pattern is similar to the carnivorous plants with passive pitcher traps (e.g. *Sarracenia, Nepenthes*) and seems to be widespread across many carnivorous taxa. The exact role of AOX in the traps of carnivorous plants remains to be investigated.

Carnivorous plants of the genus *Utricularia* capture prey using modified leaves as suction bladder traps. The bladders are hollow, water-filled vesicles with flexible lateral walls with an entrance forming a short tube which is rendered watertight by a trapdoor. The trap function is based on generating a negative hydrostatic pressure inside the trap caused by an energy-demanding water pumping process from the trap lumen to the outside. When the trap is stimulated by potential prey, or also spontaneously, the trapdoor abruptly inverts its curvature, the trap walls relax and the bladder sucks water and prey inside.^1–3^ The captured prey is then digested by an action of commensal microorganisms and endogenous plant-derived digestive enzymes.^4–6^

The bladders are active plant tissue with high metabolic rate and a high rate of mitochondrial respiration (R_D_). The high R_D_ observed in *Utricularia* bladders is probably associated with their demanding physiological functions, i.e., relating to ion and water pumping, prey digestion and nutrient absorption.^7^ Jobson and his colleagues found positively selected aminoacids (Leu-113-Ser-114 motif replaced by a radical Cys-113-Cys-114) in subunit I of cytochrome *c* oxidase (COX) across all examined *Utricularia* species.^8^ This motif lies directly at the docking point of COX helix 3 and cytochrome *c*, and modeling suggests that can alter COX/cytochrome *c* dissociation kinetics and thus the R_D_ was adaptively reinforced by these specific amino acid changes. However, plants also have an alternative route for mitochondrial electron transport. Alternative oxidase (AOX) accepts electrons from a ubiquinol pool bypassing the complex III and IV and this dramatically reduces the ATP yield of respiration.^9^ A recent study showed that carnivorous pitcher plants of the genera *Nepenthes* and *Sarracenia* have high abundances of AOX in their pitcher traps with a possible role in botanical carnivory.^10^ Because pitcher plants, in contrast to *Utricularia*, usually do not have higher R_D_ in their traps in comparison to the photosynthetic parts of the leaf,^10,11^ we were interested in studying if the AOX enzyme is also abundant in metabolically active bladders in unrelated aquatic carnivorous *Utricularia*.

We used the aquatic carnivorous plant *Utricularia reflexa* Oliver (collected in Okavango Delta, Botswana) in our experiment. *U. reflexa* produces some of the largest bladder traps within the genus (Figure 1a) and was used conveniently in other studies.^6^ The plants were grown indoors in naturally-lit 3 L aquaria with a litter of robust sedges (*Carex* sp.) used as a substrate. The water was considered oligo-mesotrophic and slightly dystrophic. Small zooplankton were added to the water to support plant growth. Adult *U. reflexa* plants were 20–30 cm long and their largest traps were 4–6 mm long. The 50 mg samples of traps, and shoots without traps, from the mature 3rd–6th leaf nodes were collected separately for Western blotting. The traps were halved to remove the water inside the bladders. Immunodetections of AOX enzyme, subunit II of cytochrome *c* oxidase (COXII) and light harvesting antennae of photosystem II (LHCB4) were performed as described previously^10^ with antibodies (cat. no. AS04 045, AS04 054, AS04 053A) purchased from Agrisera (Vännäs, Sweden). For measurements of R_D_, a Clark-type oxygen sensor was used for 12–24 separated, halved young traps 3–6 mm large from the mature 3rd–6th leaf nodes and in trap-free shoot segments of the same age.^7^ Generally, R_D_ was measured in a basic control solution of 0.1 mM NaHCO_3_ with 50 µM KCl and 25 µM CaCl_2_ in a 5.3 mL stirred chamber kept at 25.0 ± 0.1°C. The R_D_ was measured in darkness for 12–15 min. For inhibitory studies, the leaf material was blotted dry, placed in 10 ml of the solution with 5 mM SHAM or 0.5 mM KCN alone or in combination in darkness at 25°C for 3 h, and R_D_ was measured in this solution.

**Figure 1.**
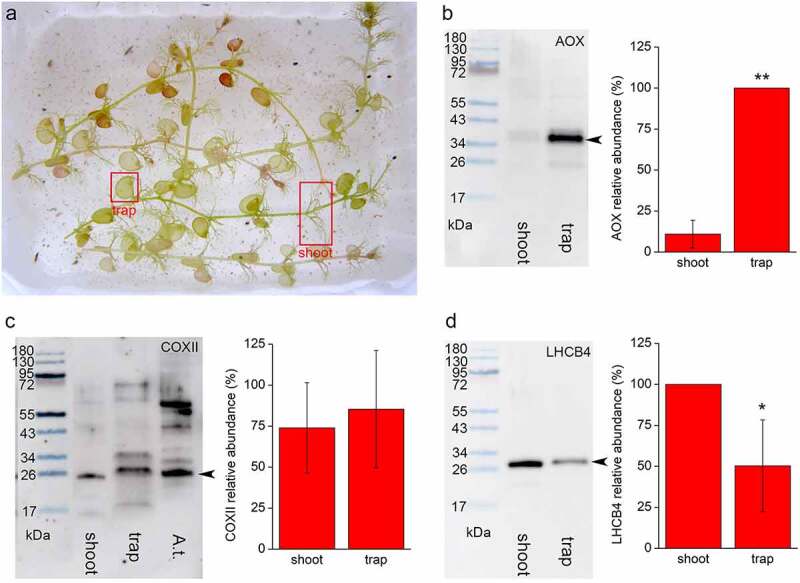
Immunodetection and quantification of AOX, COX and LHCB4 in shoots and bladder traps of *Utricularia reflexa*. (a) Plants *U. reflexa*, (b) AOX, (c) COXII, (d) LHCB4. The same amount of protein was electrophoresed under reducing and denaturing conditions, and blotted on nitrocellulose membrane. Representative blots from three independent experiments and average chemiluminescence signal intensity are shown (means ± S.D., n = 3). Arrowheads denote bands of interest. Asterisks denote significant differences at *P* < .01 (**) or *P* < .05 (*), Student’s t-test.

The abundance of AOX enzyme was 10 times higher in bladder traps in comparison to the shoots (Figure 1b). On the other hand, the abundance of COXII was more or less the same (Figure 1c). As there was a weak cross-reactivity of the COXII antibody with other proteins on the membrane, we also loaded leaf protein extract from *Arabidopsis thaliana* (A.t.) for which the epitope of the primary antibody was originally designed. Given that the bladder traps have a significantly lower chlorophyll concentration in comparison to the shoots,^7^ we also immunodetected chlorophyll-binding protein LHCB4 where we expected the opposite pattern from than in the AOX protein. Indeed, the abundance of LHCB4 in the traps was 50% of that in the shoots (Figure 1d). These results are roughly in accordance with a transcriptomic study on *U. gibba*, where *AOX* transcripts were 100-times and *COX* transcripts 10-times more abundant in the bladder traps, and transcripts of *LHCB4* in the shoots were twice as high than in the traps.^12^ The R_D_ in the bladder traps was higher in comparison to the shoots (Figure 2a,b), in accordance with a previous study^7^ and in agreement with the overall upregulation of the mitochondrial electron transport chain in bladders.^12^ To estimate the capacity of respiratory pathways, we applied inhibitors of cytochrome and alternative pathways; KCN and SHAM, respectively. KCN alone had little or no significant effect on R_D,_ indicating a high capacity of the alternative pathway in both tissues (Figure 2a). SHAM inhibited R_D_ by 49% in traps and by 36% in shoots indicating a higher AOX capacity in the traps (Figure 2b). The combined application of KCN and SHAM resulted in complete inhibition of R_D_ (0.0 ± 0.0 mmol kg^−1^ FW h^−1^ in both types of tissue). We realize that measurements of oxygen consumption rates after application of respiratory inhibitors is relative and only suitable for estimation of the capacities (maximum electron fluxes) of COX and AOX, and the true activities can only be determined by the oxygen isotope fractionation technique. Moreover, AOX is under strong post-translational control and in this case, the enzyme abundance is not a good predictor of enzyme activity.^13^

**Figure 2.**
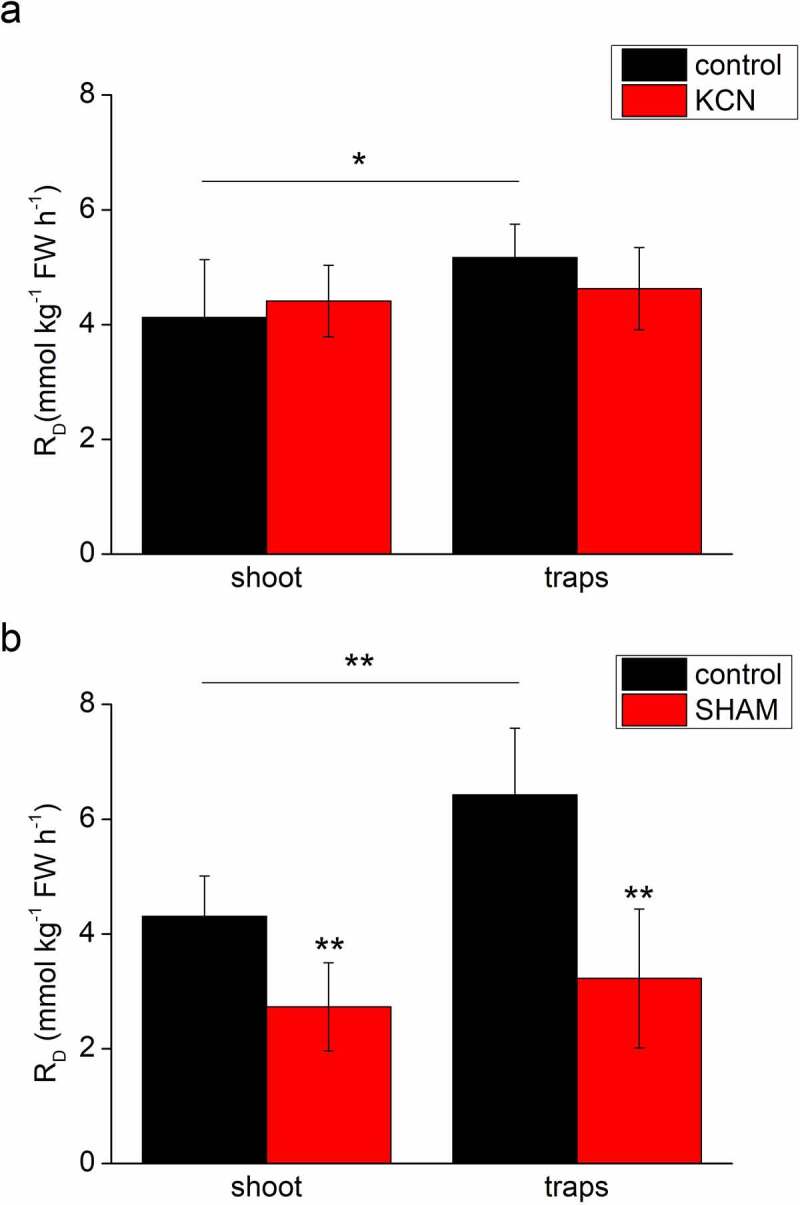
Effect of two respiratory inhibitors on respiration rate (R_D_) in shoots and bladder traps of *Utricularia reflexa*. (a) Inhibitor of cytochrome pathway, 0.5 mM KCN, (b) inhibitor of alternative pathway, 5 mM SHAM. Means ± S.D., n = 6. Asterisks denote significant differences at *P* < .01 (**) or *P* < .05 (*), Student’s t-test.

In our recent study, we discussed the possible reasons and consequences of increased AOX content in the pitcher traps of *Nepenthes*.^10^ Some of them may also hold for *Utricularia* bladders. For example, *Utricularia* bladders have also reduced A_N_ in comparison to the shoots^7^ and the possible imbalance between light and dark reactions of photosynthesis results in increased excitation pressure at photosystem II which may act as a signal for *AOX* transcription.^14^ In general, nutrient stress, which is common for all carnivorous plants, may also induce *AOX* transcription.^15^ Ammonia, a product of deamination of prey amino acids in the digestive fluid, may lead to a great increase of AOX activity and protein and transcript abundance in contrast to nitrate supply.^16^ The expression of *AOX* is also strongly induced by reactive oxygen species (ROS) through mitochondrial retrograde signaling.^17^ High production of ROS in *U. gibba* bladders as a result of high energy metabolism was recently documented.^12^

Until now, the studies on respiration of *Utricularia* have been focused mainly on COX.^8,18^ Such studies are reasonable due to the high metabolic and respiratory activities of the *Utricularia* bladders and the high demand for ATP needed for their functioning.^7^ Indeed, application of KCN strongly inhibited water exudation and trap function.^19,20^ However, our study showed very high abundance of AOX enzyme in the bladders, which is known to compete with the COX for electrons and yield only a little ATP.^13^ This finding goes against the high energy requirement for *Utricularia* bladders, but on the other hand, the AOX enzyme may have other important functions in the carnivorous organ. As a higher abundance of AOX enzyme in traps was found across different genera and orders of carnivorous plants, it seems that it is a part of the general carnivorous syndrome like reduced photosynthesis in traps.^10,11^ The exact role of AOX and its activation state in the trap organs of carnivorous plants remains to be elucidated.
